# Neural and behavioral signature of human social perception

**DOI:** 10.1038/s41598-019-44977-8

**Published:** 2019-06-25

**Authors:** Ana Saitovitch, Hervé Lemaitre, Elza Rechtman, Alice Vinçon-Leite, Raphael Calmon, David Grévent, Volodia Dangouloff-Ros, Francis Brunelle, Nathalie Boddaert, Monica Zilbovicius

**Affiliations:** 1 INSERM U1000, Department of Pediatric Radiology and IMAGINE Institute, INSERM UMR 1163, Paris Descartes University, Sorbonne Paris Cité, Hôpital Necker-Enfants Malades, Paris, France; 2 Faculté de Médecine, Paris-Sud University, University of Paris-Saclay, Paris, France

**Keywords:** Neuroscience, Social neuroscience

## Abstract

Social behavior is extremely variable among individuals, and the neural basis of this variability is still poorly understood. In this study, we aimed to investigate the neural basis of interindividual variability in the first step of social behavior, that is, social perception. For that purpose, we first used eye-tracking to measure social perception during the passive visualization of socially relevant movie clips. Second, we correlated eye-tracking data with measures of rest cerebral blood flow (CBF) obtained using arterial spin-labeling (ASL) MRI, an index of local rest brain function. The results showed a large interindividual variability in the number of fixations to the eyes of characters during passive visualization of movie clips displaying social interactions. Moreover, individual patterns remained stable across time, suggesting an individual signature of social behavior. Whole-brain analyses showed significant positive correlation between the number of fixations to the eyes and rest CBF: individuals who looked more to the eyes were those with higher rest CBF levels within the right superior temporal regions. Our results indicate the existence of a neural and behavioral signature associated with the interindividual variability in social perception.

## Introduction

Humans are, by nature, highly social beings. Although basic social perception processes and the resulting social abilities emerge from innate mechanisms, a large amount of variability between individuals can be observed^1,2^. For instance, the way individuals engage in social interactions may be very different. This variability results in a wide spectrum of social behavior, ranging from extreme shyness to extreme extroversion. Better comprehension of this interindividual variability is crucial to understanding its neural basis.

Brain imaging studies have largely investigated the neural basis of social processes. Studies using fMRI described a brain network particularly implicated in processing social information. The so-called social brain is composed of the amygdala, superior temporal sulcus (STS), orbitofrontal cortex, and fusiform gyrus^3,4^. Within this network, the STS is considered a hub for social perception and cognition, including the perception of eyes, faces and human motion, as well as understanding others’ actions and mental states^5^. These studies typically focused on communalities, averaging data across participants to reveal underlying effects despite the presence of measurement noise, rather than individualities in brain responses^6^.

More recently, brain imaging studies have started to investigate the neural correlates of interindividual variability in social functioning. For instance, results from structural MRI studies showed that altruistic behavior is strongly associated with gray matter volume in the right temporoparietal junction (TPJ)^7^. It has also been shown that higher scores of affective empathy are associated with greater gray matter density in the insula cortex, while higher scores of cognitive empathy are associated with greater gray matter density in the midcingulate cortex and dorsal medial prefrontal cortex^8^. Interestingly, individuals with poorer social cognition—namely, those least able to correctly identify facial emotion—exhibit decreased median prefrontal cortex thickness^9^. Moreover, the number of friends an individual declares on a web-based social networking service reliably predicted gray matter density in the right STS^10^.

Functional MRI (fMRI) activation studies investigating the neural basis of interindividual variability in social behavior showed that increased activation in the TPJ is associated with prosocial behavior^11^ and that individuals with high other-oriented justice sensitivity scores exhibit enhanced recruitment of the right TPJ and dorsomedial prefrontal cortex while watching bad actions^12^. In addition, it has also been shown that increased activity within the STS induced by changes in gaze direction are associated with higher scores of autistic traits in typical adults^13^. Nevertheless, as is intrinsic to fMRI paradigms, the results are inexorably task related and stimuli dependent, which prevents further information about interindividual variability within rest brain function.

In this context, resting state fMRI (rs-fMRI) studies have demonstrated that intrinsic brain functional connectivity is associated with interindividual variability in cognitive performances. Functional connectivity has been associated with individual differences in cognitive profiles, such as IQ, musical ability and reading ability^14^. Moreover, individual-specific network topography has also been associated with phenotypes across personality and emotion^15^. For instance, the characteristics of functional networks can predict the five-factor personality traits. Connectivity between the precuneus and brain areas involved in self-evaluation, such as the dorsomedial prefrontal cortex, are associated with neuroticism; connectivity between the anterior cingulate and precuneus with brain areas involved in reward and motivation, such as the lateral paralimbic regions, are associated with extraversion^16^. In addition, resting-state connectivity between the middle orbito-frontal cortex and putamen has been associated with impulsivity traits^17^.

Although brain imaging investigations of the neural basis of interindividual variability in social processes have become a large field in the past decade, methodological challenges still exist. Indeed, a main challenge concerns objectively measuring such variability since most studies rely on a questionnaire-based approach or self-reported measures, which remain highly subjective, often unreliable and are a major sources of self-reporting bias^18^. Therefore, in the present study, we aimed to investigate the neural basis of interindividual variability in the first step of social behavior, that is, social perception. Specifically, we tested two hypotheses: (1) it is possible to objectively measure interindividual variability in social perception using eye-tracking, and (2) this putative interindividual variability is correlated with individual differences in local rest brain function.

For that purpose, two studies were conducted. In Study 1, we used eye-tracking to measure interindividual variability of gaze behavior, an objective index of social behavior, focusing on the eye as a key region for social perception. Gaze to the eyes was measured during passive visualization of naturalistic social movies to elicit the most automatic spontaneous behavior^19–21^.

In Study 2, we investigated in an additional non-overlapping group whether the interindividual variability in this basic mechanism of looking to the eyes is associated with an individual pattern of local rest brain function measured by arterial spin labeling (ASL) MRI. Indeed, recent advances in the brain-imaging field allow quantitative measures of rest cerebral blood flow (CBF) using MRI. ASL-MRI is an imaging technique used to quantitively measure rest CBF, a direct index of local synaptic activity, noninvasively by magnetically labeling inflowing blood. Importantly, eye-tracking measures and rest CBF measures were obtained independent of each other, since acquisition was not simultaneous.

## Methods

### Participants

Forty-seven healthy volunteers participated in this study. Fourteen subjects (mean age = 25.86 ± 4.94 years; seven women) participated in Study 1, undergoing seven repetitive visualizations of the eye-tracking stimuli. Thirty-three non-overlapping subjects (mean age = 22.3 ± 2.8 years; four women) participated in Study 2, undergoing a single visualization of the eye-tracking stimuli and an MRI. All participants were right-handed, had normal or corrected-to-normal sight and were free of psychiatric, neurological and general health problems. All participants were monetarily compensated for their participation in this study and provided written informed consent. All methods were performed in accordance with the relevant guidelines and regulations. This study was approved by the Ethics Committee of the Saint Louis Hospital, Paris, France.

### Eye-tracking protocol

The study was performed using the Tobii T120 eye tracker equipment, based on infra-red technology, consisting of a 17-inch TFT monitor with a resolution of 1280 × 1024 pixel, from which the stimuli were presented in full screen, and the gaze behavior was simultaneously recorded. The eye-tracking system was completely non-invasive with little indication that the eye movements were being tracked and with no artificial constraints of the head or body movements. The system tracked both eyes to a rated accuracy of 0.5 degrees with a sampling rate of 60 Hz. The Tobii equipment was connected to an HP Pavillon dv6 laptop computer (Windows 7 Professional).

The participants were individually tested and were seated facing the eye-tracker monitor at a distance of approximately 60 cm; the experimenter sat next to the participant to control the computer without interfering with the viewing behavior. A calibration test consisting of five registration points was performed before each set of stimuli. The calibration test was repeated if the examiner considered one of the five points not valid according to the eye-tracker criteria (recorded gaze extrapolating the limits of the calibration-designed area or absence of recording for one of the five points). All participants matched general recording quality criteria, based on the amount of valid and missing data, as indicated by Tobii Studio software.

The participants were instructed that they would see a sequence of clips and all they had to do was watch them. Since we have used stimuli composed of short clips extracted from a popular French commercial film (Le Petit Nicolas), at the end of the eye-tracking sessions, participants were asked if they were familiar with the presented film clips and confirmed have seen it once. The stimuli creation, the calibration procedures and the data acquisition and visualization were performed using the Tobii Studio software.

### Stimuli

The stimuli set was composed of short clips extracted from a French commercial film (25 fps). A total of six clips of 10 sec each displaying social scenes with two characters engaged in peer-to-peer social interactions (Le Petit Nicolas) were selected and assembled together creating a final movie of 60 sec.^22^. No specific task performance was required. Factors such as scene background or character position were not controlled for. The choice of the stimuli was deliberate to produce the most ecological and naturalistic stimuli set, since previous studies have shown that the most spontaneous automatic gaze behavior is elicited by dynamic stimuli presenting real characters^19,20^.

### Autism-Spectrum Quotient

To investigate rest functional brain correlates of a standardly used measure of social behavior, Autism-Spectrum Quotient (AQ) questionnaire was collected from all participants. This self-administrated scale intents to identify the degree to which any individual adult with normal IQ may have “autistic traits” (Baron-Cohen *et al*., 2001). The AQ consists of 50 items, each of which is in a forced choice format, assessing personal preferences and habits. Subjects rated to what extent they agree or disagree with the statements. Responses considered in line with autistic traits are scored 1, while the other ones are scored 0. For approximately half the items an “agree” response is in line with autistic traits (e.g. item 23: “I notice patterns in things all the time”); for the other half a “disagree” response is indicative of an autistic trait (e.g. item 11: “I find social situations easy”). All the item scores are summed and higher AQ total score indicate higher presence of autistic traits.

### Arterial Spin Labeling (ASL)

Arterial spin labeling (ASL) is a noninvasive MRI method that uses magnetically labeled blood water as a flow tracer, providing rest CBF images of the brain. In ASL, the diffusible tracer is a magnetic label applied to blood water molecules produced by saturating or inverting the longitudinal component of the MR signal. If all of the label arrives at the capillary bed or tissue at the time of imaging, this results in a T1-weighted signal reduction proportional to CBF, called the tagged image; the tagged image is compared to a control image in which the blood water molecules are not perturbed^23^. Moreover, ASL-MRI also provide an absolute, quantifiable CBF measurement on a voxel-by-voxel basis. Finally, ASL-MRI has a short acquisition time, does not require radioisotope injections^24^.

### MRI scans

All MRI exams included T1-weighted and ASL sequences and were acquired on a General Electric Signa 1.5 T MRI scanner in the Necker Hospital. We acquired a 3D pseudocontinuous arterial spin labeling (3D pcASL) sequence using a fast spin echo acquisition with spiral filling of the K space (TR/TE: 4453/10.96 msec, 8 spiral arms x 512 sampling points, post-labeling delay: 1025 msec, flip angle: 155°, matrix size: 128 × 128, slice thickness: 4 mm, Field of View: 24 × 24 cm, 40 contiguous axial slices, duration: ≈ 5 mins). CBF quantification from ASL tagged and control images was performed and warranted by General Electric 3D ASL software (Zaharchuk G, 2009). Anatomical sequences were used only for spatial normalization of ASL sequences.

## Statistical Analysis

### Eye tracking

In each movie clip, areas of interest (AOIs) were defined around the eyes of the characters. The number of fixations in each AOI was recorded using the Tobii Studio software. A fixation event was defined by the Tobii fixation filter based on a 0.42 pixels/ms threshold. The number of fixations was calculated for all participants. The number of fixations to the eyes was selected since it is an absolute variable that informs exploratory behavior towards a defined region: a higher number of fixations indicates that people are likely to further explore the region^25^. Importantly, the number of fixations to the eyes and total amount of time spent looking at the eyes are strongly correlated (R = 0.79; *p* = 4.206e-08). In both studies, the number of fixations to the eyes were pooled together from all clips providing an individual score of the number of fixations to the eyes.

In Study 1, a repeated measure ANOVA was performed to study the impact of repeated visualization of the stimuli on gaze pattern; the number of fixations to the eyes for each participant for each of the seven visualizations was used as the repeated factor. In addition, a Shapiro-Wilk normality test was performed to investigate the distribution of eye-tracking data, namely, individual scores of the number of fixations to the eyes.

### ASL-MRI

Structural T1-weighted and ASL images were analyzed using SPM8 (http://www.fil.ion.ucl.ac.uk/spm) implemented in Matlab (Mathworks Inc., Sherborn, MA, USA). Briefly, structural T1 images were segmented into grey matter, white matter and cerebrospinal fluid using the VBM8 toolbox (http://dbm.neuro.uni-jena.de/vbm/). The ASL images were co-registered to the corresponding native grey matter images and spatially normalized to the MNI space using the deformation matrices from the segmentation process. The resulting ASL images were smoothed using an isotropic Gaussian filter of 8 mm.

In Study 2, whole-brain, voxel-by-voxel statistical analyses were performed using a mass-univariates approach within the classical framework of the general linear model of SPM8. This approach provided an appropriate design to perform correlation analyses on the smoothed and normalized ASL images. The number of fixations to the eyes obtained from eye-tracking measures for each subject, or individual AQ scores, were entered as independent variables within the framework of the general linear model within SPM8. The analyses were constrained to gray matter tissue only by thresholding the mask of analysis to 50% of the mean gray matter image of our sample. P values were set to 0.05 Family Wise Error (FWE) corrected for multiple comparisons.

## Results

### Study 1. Eye-tracking study

**Figure 1:**
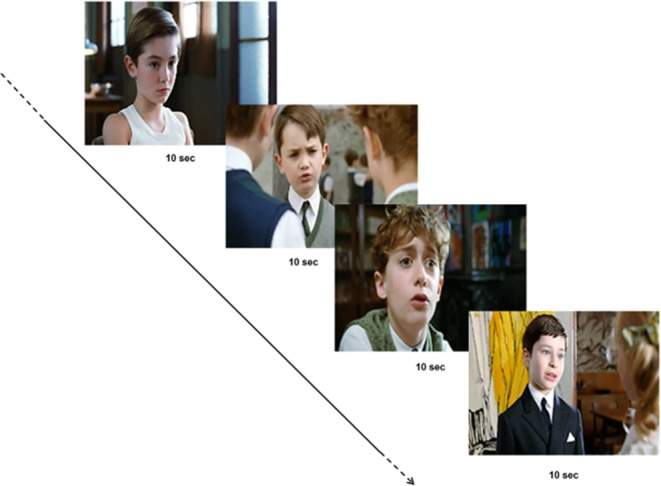
Example of eye-tracking stimuli set: a total of six clips of 10 sec each displaying social scenes with two characters engaged in peer-to-peer social interactions (Le Petit Nicolas) were selected and assembled together to create a final movie of 60 sec.

**Figure 2:**
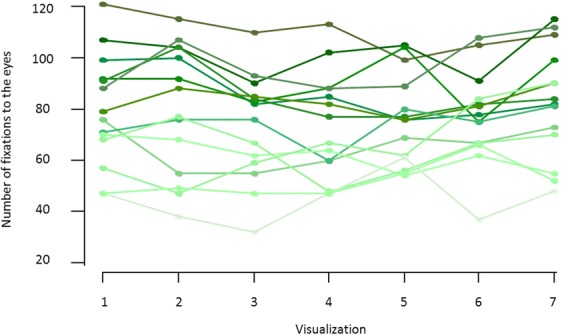
Individual plots of the number of fixations to the eyes over the seven visualizations from 14 young healthy volunteers participating in Study 1 (each color represents one participant). The plot shows great between-subject variability as well as stability in the individual gaze patterns across time.

To investigate the interindividual variability in gaze behavior, we used eye tracking to measure gaze patterns in a group of 14 young healthy volunteers during visualization of a set of stimuli presenting naturalistic social movies, namely, characters engaging in social interactions (Fig. 1). As illustrated in Fig. 2, the number of fixations to the eyes largely differed among participants (mean = 79.5 ± 21.7; range = 47–121), indicating great variability in gaze patterns in healthy young volunteers when perceiving social stimuli. Furthermore, to investigate the stability of gaze patterns across time, participants watched the same stimuli set seven successive times. Using a linear regression analysis with repeated measures, we showed no impact of successive visualizations on the number of fixations to the eyes (F_1,13_ = 0.27; p = 0.60), indicating within-subject stability in gaze pattern across time (Fig. 2).

### Study 2. Brain imaging study - correlation with eye-tracking data

To investigate whether the interindividual variability in social perception correlated with individual patterns of local rest brain function, we studied an additional non-overlapping group of 33 young healthy volunteers. Participants performed an eye-tracking session, and gaze pattern was measured using the same stimuli set described in the previous study (Fig. 1). In addition, participants underwent an MRI in which ASL-MRI was used to measure whole-brain CBF at rest, an index of local rest brain function.

**Figure 3:**
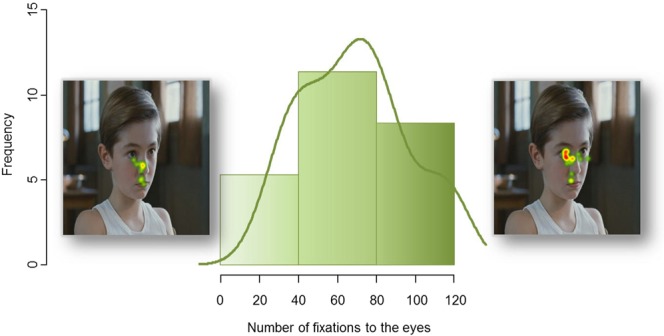
Frequency histogram of the number of fixations to the eyes from the 33 healthy volunteers participating in Study 2. This confirms great between-subject variability in this behavior and its normal distribution.

**Figure 4:**
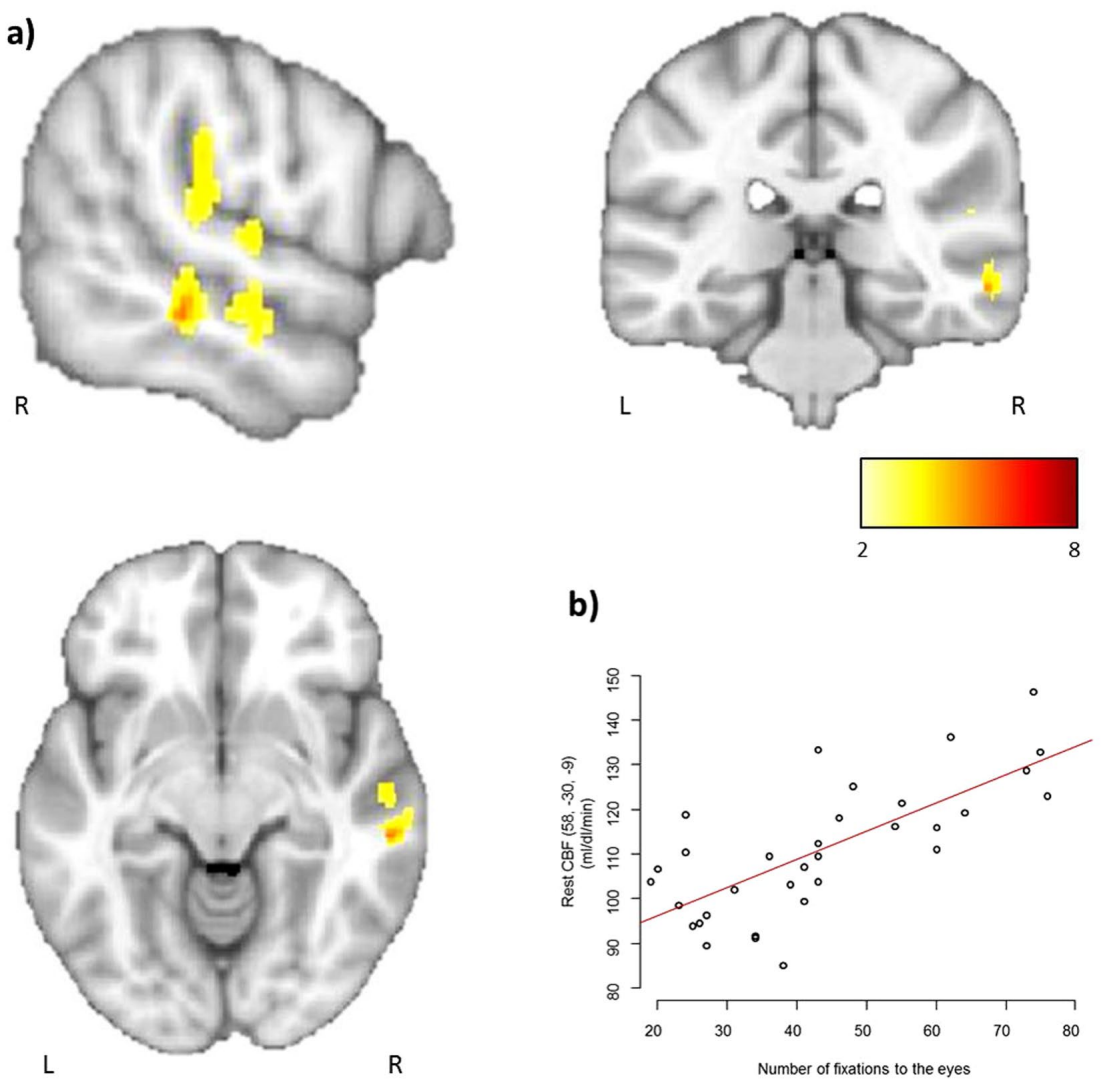
(**a**) Results of whole brain correlation analysis between the number of fixations to the eyes, measured by eye-tracking, and rest CBF, measured by ASL-MRI, overlaid on the MNI-152 template average brain (for illustration purposes, p values were set to 0.001 uncorrected). The MNI coordinates of the two significant peaks at p < 0.05 Family Wise Error (FWE) corrected for multiple comparisons at both the voxel and cluster level: x = 51, y = −25, z = 22; T = 5.89; P_FWE_ = 0.009; k_E_ = 48, P_FWE_ = 0.008 and x = 58, y = −30, z = −9; T = 5.47; P_FWE_ = 0.024; k_E_ = 3, P_FWE_ = 0.038. The color bar represents T-scores. (**b**) Plot of the correlation between the number of fixations to the eyes and the individual rest CBF values.

Analyses of the eye-tracking data in this second independent group confirmed a large interindividual variability in the number of fixations to the eyes (mean = 68.6 ± 27.1; range = 94). Interestingly, the variation in the number of fixations to the eyes followed a normal distribution (W = 0.95; p = 0.19) (Fig. 3). Whole-brain, voxel-by-voxel correlation analyses, performed within SPM, between the number of fixations to the eyes and rest CBF showed a significant positive correlation (p < 0.05 FWE corrected for multiple comparisons) exclusively located in the right superior temporal region. Participants who looked more to the eyes of characters during visualization of the social movies had significantly higher cerebral blood flow at rest in the right temporal region, mainly the superior temporal sulcus (STS) (Fig. 4).

In addition, in order to investigate the rest functional brain correlates of a standardly used measure of social behavior, the 33 subjects completed the self-administered questionnaire on autistic traits (AQ)^26^. Whole-brain voxel-by-voxel correlation analyses between individual AQ scores and rest CBF were performed, and no statistically significant results were observed.

## Discussion

To the best of our knowledge, this is the first study that investigated typical interindividual variability in gaze behavior during visualization of social scenes, which is an objective index of social engagement, and its association with local rest brain function. Our results indicate major differences between subjects’ behaviors in this fundamental stage of social cognition. When viewing social scenes, some individuals look more to the eyes of characters while others look less, all remaining within the spectrum of typical social functioning. In addition, as we have hypothesized, objectively measuring interindividual differences in this basic step of social behavior reveals correlations with rest brain function within the STS.

These results provide new insight into understanding interindividual variability within broader social behavior. Eye gaze behavior is associated with broader aspects of social behavior. Indeed, the ability to process eye gaze information is negatively correlated with self-reported loneliness: individuals who show poorer abilities to detect gaze information have higher scores on the self-reported loneliness scale^27^. In addition, eye-tracking studies have shown that visual patterns while watching emotionally loaded images may depend on personality traits^28,29^. Here, rather than describing variability based on subjective measures of complex social behavior, our results point towards interindividual differences in a basic process that is already present from an early stage of development and thus is a key component of social behavior. Interestingly, our results do not show significant correlation between the AQ, a questionnaire largely used to measure social behavior, and brain functioning at rest. This indicates that eye-tracking measures of social perception might be more sensitive at objectively identifying differences between subjects in social behavior and their correlates to rest functional properties of the brain.

The importance of gaze perception in broader social interactions has been largely established. Eye contact helps infer the intentions and feelings of the conspecifics, which is crucial for survival and social integration^30^. Moreover, the preference for the eyes as a privileged attention target is evident extremely early in the normal development, suggesting that this preference is a core mechanism for the subsequent development of a larger expertise of human social cognition^31^. Even though gaze perception abnormalities have been described in different psychiatric disorders^21,32–34^, the normal variability in social perception outside the scope of pathology has rarely been addressed. Our findings indicate not only that there is a great variability in how healthy individuals look at the eyes, and that it can be objectively measured using eye-tracking, but also that there is as a large intraindividual stability. This suggests the existence of an individual signature regarding social behavior.

Repeated visualization of identical stimuli could engender a habituation effect. Our results do not show such effect throughout seven consecutive visualizations of the same stimuli set. The absence of habituation is probably due to the characteristic of the stimuli, composed of very short (10 sec) movie clips. This confirms results from a previous study conducted in our lab^22^. The lack of habituation during repeated visualization of the stimuli is an additional new finding that corroborates our hypothesis of an individual signature on social behavior. Interestingly, recent work from Ami Klin and colleagues showed that monozygotic toddlers present high concordance in levels of eye-looking, measured with eye-tracking during visualization of naturalistic social movies^35^. Such findings, in line with the present results, suggest that social visual engagement constitute a neurodevelopmental endophenotype for interindividual variability in social behavior.

Taking a step further towards understanding the neural basis of social behavior, our results indicate the existence of a neural signature associated with interindividual variability in social perception. Interestingly, this correlation is observed within the right posterior STS. Individuals who looked more to the eyes of characters during passive visualization of naturalistic social scenes exhibited higher rest CBF values within the right posterior STS, and vice-versa. These results suggest that levels of brain activity at rest in a precise circumscribed region in the brain can predict the number of fixations to the eyes.

As the STS is thought to have a major role in procession social information, an individual signature of social behavior associated with a neural signature of brain functioning bounded to the STS becomes increasingly relevant^3,5,36^. Indeed, the STS is implicated not only in the perception of biological motion^37,38^ but also in the perception of the eyes^39–41^, which is a key mechanism of social behavior, as mentioned above. Notably, in social developmental pathologies such as autism spectrum disorders, both anatomo-functional abnormalities within the STS and deficits in eye-gaze perception have been described^42,43^.

Given the importance of social interactions for humans, it is not surprising that most psychiatric disorders involve some disruption of normal social behavior; further, abnormal social functioning is one of the central symptoms in several disorders such as autism, social anxiety disorder, and borderline personality disorder^44^. Despite the importance of social interactions, our understanding of the neural factors implicated in social behavior is still limited. Moreover, until recently, interindividual variability was usually regarded as a source of noise for research.

The neural correlates of normal variation in social behavior have recently become a hot topic within the field of social neuroscience. Together, the results indicate the existence of a “spectrum of normality” in social behavior and its brain correlates. Nevertheless, few investigations have focused on local rest brain correlates to date. Modern neuroimaging methods such as ASL-MRI enable relation of individual differences in social perception to the underlying baseline brain mechanisms. ASL-MRI technique, compared to those using BOLD signal, has the advantage of providing an absolute quantification of rest CBF. In addition, ir provides increased spatial specificity to neuronal activity due to the capillary/tissue origin of the ASL signal^45^. This novel approach contributes to a growing body of research in this field and can complement current resting-state connectivity literature to help understanding interindividual variability in social behavior.

It should be mentioned that in this study, we deliberately focused on the precise behavior of looking to the eyes, since it has been shown to be the first step to broader social interactions^46^. Further investigations may focus on broader aspects of visual behavior by studying more complex scan paths, for instance.

In this study, we have shown that rest CBF levels within the STS predicts individual gaze behavior in a social context. We have therefore demonstrated, for the first time, an association between local rest brain function and an objective measure of social behavior variability in young healthy volunteers. This suggests the existence of an individual signature in both social behavior and local rest brain function.
